# Efficiency and safety of inhalative sedation with sevoflurane in comparison to an intravenous sedation concept with propofol in intensive care patients: study protocol for a randomized controlled trial

**DOI:** 10.1186/1745-6215-13-135

**Published:** 2012-08-10

**Authors:** Jens Soukup, Antje Selle, Andreas Wienke, Jörg Steighardt, Nana-Maria Wagner, Patrick Kellner

**Affiliations:** 1 University Clinic for Anaesthesiology and Operative Intensive Care Medicine Martin-Luther-University Halle-Wittenberg, Halle/Saale, Germany; 2 Institute of Medical Epidemiology, Biostatistics, and Informatics, Martin-Luther-University Halle-Wittenberg, Halle/Saale, Germany; 3 Coordination Centre for Clinical Trials, Martin-Luther-University Halle-Wittenberg, Halle/Saale, Germany; 4 Clinic for Anaesthesiology, Intensive Care Therapy and Palliative Medicine, Carl-Thiem-Clinic Cottbus, Cottbus, Germany; 5 Clinic for Anaesthesiology and Critical Care Medicine, University Hospital Rostock, Rostock, Germany

**Keywords:** Inhalative sedation, Intravenous sedation, Intensive care, Sevoflurane

## Abstract

**Background:**

State of the art sedation concepts on intensive care units (ICU) favor propofol for a time period of up to 72 h and midazolam for long-term sedation. However, intravenous sedation is associated with complications such as development of tolerance, insufficient sedation quality, gastrointestinal paralysis, and withdrawal symptoms including cognitive deficits. Therefore, we aimed to investigate whether sevoflurane as a volatile anesthetic technically implemented by the anesthetic-conserving device (ACD) may provide advantages regarding ‘weaning time’, efficiency, and patient’s safety when compared to standard intravenous sedation employing propofol.

**Method/Design:**

This currently ongoing trial is designed as a two-armed, monocentric, randomized prospective phase II study including intubated intensive care patients with an expected necessity for sedation exceeding 48 h. Patients are randomly assigned to either receive intravenous sedation with propofol or sevoflurane employing the ACD. Primary endpoint is the comparison of the ‘weaning time’ defined as the time required from discontinuation of the sedating agent until sufficient spontaneous breathing occurs. Moreover, sedation depth evaluated by Richmond Agitation Sedation Scale and parameters of patient’s safety (that is, vital signs, laboratory monitoring of organ function) as well as the duration of mechanical ventilation and overall stay on the ICU are analyzed and compared. An intention-to-treat analysis will be carried out with all patients for whom it will be possible to define a wake-up time. In addition, a per-protocol analysis is envisaged. Completion of patient recruitment is expected by the end of 2012.

**Discussion:**

This clinical study is designed to evaluate the impact of sevoflurane during long-term sedation of critically ill patients on ‘weaning time’, efficiency, and patient’s safety compared to the standard intravenous sedation concept employing propofol.

**Trial registration:**

EudraCT2007-006087-30; ISCRTN90609144

## Background

Present guidelines for analgosedation on intensive care units (ICU) of the Society of Critical Care Medicine (SCCM) as well as the German Society for Anaesthesiology and Intensive Care Medicine (DGAI) currently favor intravenous sedation concepts employing propofol for sedation up to 72 h and midazolam for long-term sedation [1]. However, application of either substance is associated with serious adverse effects such as the negative influence of propofol on hemodynamics or propofol infusion syndrome as well as ceiling effects or the risk of accumulation with incalculably prolonged wake-up times related to long-term use of benzodiazepines, respectively [2-5].

The clinical implementation of volatile anesthetics for long-term sedation of specific patients on intensive care units has already been reported in the late 1980s. In particular, benefit from inhalative sedation was initially suggested for patients with bronchial asthma or those requiring the combination of a variety of hypnotics and analgesics for adequate analgosedation (that is, patients with drug abuse or addiction syndromes) [6]. Indeed, volatile anesthetics provide the advantage of safe sedation at the same time as increased controllability compared to most intravenous sedation agents as they lack accumulation or tolerance development. In this regard, previous studies provide evidence for essentially shorter and more predictable wake-up times of patients sedated by inhalation compared to those sedated by propofol [7]. While a reduction in the time needed for the recovery of alertness and sufficient spontaneous breathing allows for immediate evaluation of neurological status, it also reduces the time of mechanical ventilation and ventilation-associated complications thus leading to a shorter duration of the requirement for treatment of patients on the ICU [8-12]. Moreover, volatile anesthetics such as sevoflurane itself have been shown to exert organ protective, that is cardioprotective effects, which render patients with cardiovascular disease specifically eligible for potential benefit from sedation by inhalative agents [13,14].

As the application of volatile anesthetics requires a classical vaporizer combined with a ventilation device or the use of specifically designed so-called ‘closed anesthesia systems’, inhalative sedation was mainly restricted to operation rooms and thus not applicable on intensive care units so far. However, with the invention of the anesthetic-conserving device (ACD, AnaConDa®, Sedana Medical, Uppsala, Sweden), a technical device has become available which enables safe application in clinical daily routine on the ICU by providing implementable size of the device itself at the same time as avoidance of ambient air contamination [8,9,11]. Due to their physicochemical characteristics, only sevoflurane and isoflurane can be applied as volatile anesthetics with the AnaConDa® system. Because of their low vapor pressure, the use of a specific vapor is not required and both isoflurane and sevoflurane can be mixed in their liquid aggregation state into the breathing gas via a perfusor integrated in the ACD.

In the present study, we aimed to compare the standard use of intravenous sedation with propofol with inhalative sedation employing sevoflurane applied by the ACD in mechanically ventilated patients requiring sedation for longer than 48 h on the ICU. Primary endpoint in this currently ongoing, two-armed, monocentric randomized prospective phase II study is the time required to re-establish sufficient spontaneous breathing and extubation following discontinuation of the sedative medication. Moreover, parameters such as sedation depth, changes in hemodynamic and chemical laboratory parameters, overall ventilation time and frequency of ventilator-associated pneumonia, myocardial ischemia as well as the overall duration of the ICU stay and the related process costs are closely monitored, analyzed, and compared. The results derived from this trial may therefore help to dissect the future perspectives of inhalative long-term sedation with volatile anesthetics such as sevoflurane with respect to efficiency and patient’s safety on intensive care units.

## Method

### Study design

The trial was designed as a two-armed, monocentric, randomized prospective phase II study for long-term (that is, >48 h) sedation of intensive care patients. A novel sedation regime employing sevoflurane applied by inhalation is compared to the commonly used intravenous procedures with propofol that are in accordance with current guidelines for sedation of intensive care patients.

### Patient characteristics

Eligibility of patients was verified in accordance to the inclusion and exclusion criteria given below. Having obtained informed written consent of the patient or the patient’s legal representative for participation in the clinical trial, randomization and allocation to a therapy group is carried out.

#### Inclusion criteria

 – Intubated patients with expected necessity for long-term sedation > 48 h

 – Age ≥ 18 years

 – Signed declaration of consent by the patient or a legal representative

 – Continuous mechanical ventilation for less than 48 h by the time of recruitment

#### Exclusion criteria

 – No indication for sedation

 – Pregnancy and breastfeeding

 – Acute pulmonary failure

 – Existent severe liver failure

 – Primary poor prognosis

 – Participation in another study during the previous 30 days

 – Contraindications against the study medication

#### Interventions to be evaluated

**Figure 1:**
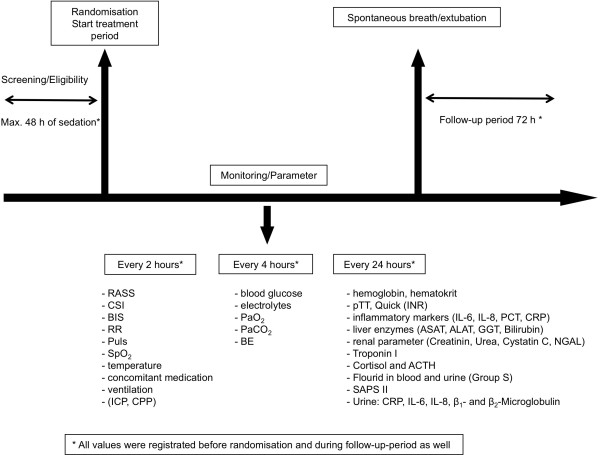
Flowchart of the study set-up.

Following assurance of eligibility according to the inclusion and exclusion criteria, patients are randomly assigned to either the intervention group to receive sevoflurane using the ACD (Group S) or the control group receiving propofol followed by midazolam on day 4 (Group P). A flowchart of the study is depicted in Figure 1.

### Monitoring of sedation

**Figure 2:**
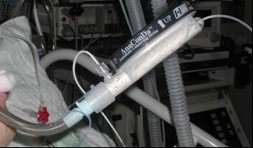
**The anesthetic-conserving device (AnaConDa**®**) in the clinical set-up.**

Patients of group S are sedated by continuous application of sevoflurane employing the anesthetic-conserving device (ACD, AnaConDa®, Sedana Medical, Sweden). This anesthesia gas-recirculation-system consists of a miniature vaporizer integrated into the ventilation hose system between the Y-piece and the patient, thereby replacing conventional ventilation filters (Figure 2). In addition to a conventional heat and moisture exchange (HME) filter, the miniature vaporizer comprises a lipophilic-activated carbon particle filter absorbing the majority of exhaled anesthetic condenses. The system-specific ‘dead space’ is 100 mL. The detailed functional principle of the system has already been described in detail [15-18].

Based on current guidelines, sedation of patients in group P is initially conducted by intravenous application of propofol (Disoprivan 2%). On the 4^th^ day of sedation, propofol is changed to midazolam in order to avoid propofol infusion syndrome. To ensure adequate analgetic co-regimes, both study groups are subjected to continuous application of remifentanil or sufentanil.

Sedation depth is monitored by determination of to the Richmond-Agitation-Sedation Scale (RASS) (pursuing a target score value of −3 or −4) every second hour as well as continuous monitoring of the Bispectral Index (BIS) and the Cerebral State Index (CSI) aiming for index values of 40 ± 10.

During sedation, all vital parameters including blood pressure, heart rate, temperature, and pulsoximetry are continuously monitored and calibration of the respirator is ensured. In patients with severe neurological damage, intracranial pressure (intraparenchymal tube, external liquor drainage) and cerebral perfusion pressure are additionally monitored. Arterial blood gas analysis is performed every 4 h in order to exclude significant deviations in acid–base metabolism, electrolyte balance, or levels of blood glucose. Laboratory parameters such as blood counts, hemostasis, parameters of infection, as well as indicators of hepatic and renal function are analyzed on a daily basis. In addition, serum levels of fluoride, cortisol, ACTH, and Troponin I are determined (Figure 1).

### Duration of study

The observation period of the study lasts until 3 days after extubation or when spontaneous breathing is persistent via tracheostomy. The planned overall duration of the clinical trial will be 24 months.

### Primary endpoint

Primary endpoint of the study is the time required from discontinuation of application of the sedating agent (that is, sevoflurane or propofol) until extubation or persistent spontaneous breathing is possible via tracheostomy (‘weaning time’).

In this regard, the following events in the wake-up phase are documented in order of appearance:

O Opening of eyes

O Ability to exert purposive motor function

O Achievement of the ability to cooperate, that is to follow instructions

O Beginning of spontaneous breathing and subsequent extubation

## Secondary endpoints

### Evaluation of sedation quality

Secondary endpoint is the quality of sedation determined by the ratio of the targeted sedation depth to the actual sedation depth measured by RAS-score. In group S, the number of adjustments of exhalative sevoflurane concentration is registered, while in group P the necessity for supplementary bolus injections is enumerated.

### Safety

All vital signs such as blood pressure, heart rate, and peripheral oxygen saturation (SpO_2_) are monitored continuously. Blood gas analyses are performed every fourth hour and chemical laboratory parameters as well as urea, creatinine, cystatin C, interleukin 6 and 8 (IL-6, IL-8), C-reactive protein (CRP), procalcitonin (PCT), coagulation parameters, blood count, aspartate-aminotransferase (ASAT), alanine-aminotransferase (ALAT), gamma-glutamyl-transferase (GGT), bilirubin, and troponin I are determined at least on a daily basis. The course of adrenocorticotropic hormone (ACTH) and cortisol serum levels is monitored by daily measurements in order to detect the influence of the respective trial substance on the neuroendocrine system.

Determination of fluoride concentrations in blood and urine is performed in order to rule out substance-associated effects on renal function. Also, Neutrophil Gelatinase-Associated Lipocalin (NGAL) and urine proteins (CRP, IL-6, IL-8, ß-1, and ß-2 microglobulin quantitatively) are also measured daily (Figure 1).

Moreover, criteria for patient safety including duration of mechanical ventilation, incidence of ventilation-associated pneumonia, newly developed myocardial ischemia, and frequency of acute renal failure are evaluated separately for groups P and S. Finally, the length of the overall stay on the ICU and the daily Simplified Acute Physiology Score II (SAPS II) are documented for each study patient.

### Economical considerations

The costs of consumables and nursing per patient will be documented by means of the LEP® nursing intervention and workload measurement system.

## Statistical methods

### Estimation of sample size

Calculations of sample size for the propofol group (group P) are based on the assumption of a median weaning time of 132 ± 258 min following long-term sedation in ICU patients with propofol as previously described [19]. Regarding the sevoflurane group (group S), unpublished observations from 14 intensive care patients in our clinic revealed a median weaning time of 22.8 ± 6.7 min. Thus, a sample size of 47 patients per group will be necessary for a two-armed t-test, a first order error of α = 0.05, and a power of 80%. With respect to a planned drop-out rate of 5%, the necessary sample size will be 50 patients per group.

### Final evaluation

An intention-to-treat analysis (ITT) will be carried out as the standard evaluation method with all patients for whom weaning time can be determined. In addition, a per-protocol analysis will be carried out in order to analyze those patients with unforeseeable changes of sedation regimes compared to the regime they were initially randomized to.

The ITT analysis serves for the confirmatory decision-making. In contrast, the peer protocol analysis is used to describe the puristic substance-related effect and may further help to separate the effects of each sedation concept itself. However, this approach does not always reflect the everyday clinical practice.

The time required from discontinuation of application of the sedating agent (either sevoflurane or propofol) until extubation or persistent spontaneous breathing via tracheostomy (weaning time) will be analyzed employing Student’s t-test or Mann–Whitney U-test for absence of normal distribution, respectively. Moreover, the number of assessments of actual sedation depth revealing the identical sedation depth as pursued with reference to the RAS score as well as ventilation time, length of ICU stay, and the related costs will be compared. The two binary secondary criteria, namely ventilation-associated pneumonia and myocardial ischemia, will be evaluated using the chi-square test. All other secondary criteria will be presented descriptively with respect to the time course of each individual patients study period.

### Ethical considerations

The trial has been registered in a public trials registry (registry number ISRCTN 90609144). The trial will be conducted according to the principles of good practice (ICH-GCP) and the Data Protection Act 1998 and the actual laws in Germany (German drug law, German data protection law) [20,21]. The trial is sponsored by the Martin-Luther University of Halle. The Trial Management Group, through the Trial Steering Committee, will ensure that adequate systems are in place for monitoring the quality of the study (compliance with Good Clinical Practice (GCP)) and expedited (when appropriate) and routine reports of adverse effects.

The study protocol at hand has been positively reviewed by the responsible ethics commission of the Medical Faculty of the University Halle. This study primarily recruits patients that are not able to give consent. According to §41 (3) of the German Medicinal Products Act (‘Arzneimittelgesetz’ AMG) ‘…the use of the investigational medicinal product must be indicated, according to the findings of medical science, in order to save the life of the person concerned, to restore him or her to health or to alleviate suffering. Furthermore, such research must relate directly to a life-threatening or highly debilitating clinical condition suffered by the person concerned and the clinical trial may involve as little burden and other foreseeable risks as possible for the person concerned. Both the degree of burden and the risk threshold must be defined specifically in the trial protocol and monitored constantly by the investigator. The clinical trial may only be conducted if there is a justified expectation that the benefits of using the investigational medicinal product for the person concerned outweigh the risks or that the use does not entail any risks…’ [22].

The AnaConDa®-system itself as well as the required residue gas filters are CE-certified medical products. These systems are currently in clinical use, though the study situation is relatively limited in comparison to the conventional concepts established to date. Sevoflurane is a volatile hypnotic that is routinely used in anesthesia. According to its technical information sheet pursuant to § 11a AMG, the substance is suited for initiation and preparation of inhalative anesthesia of grown-ups and children, on an outpatient as well as inpatient basis. Possible problems result from the relatively sparse experience in long-term use. Therefore, this clinical study brings together comprehensive daily laboratory evaluations in order to detect clinically relevant side effects on the heart, liver, and kidneys as soon as possible. Finally, the results of the present study may help to provide evidence regarding parameters related to patient safety and the occurrence of adverse effects when sevoflurane and propofol are compared and may thus support dissecting the potential role of inhalative sedation concepts in the future.

Initial results from the employment of the AnaConDa®-system, particularly with isoflurane, have provided evidence for shorter mechanical ventilation duration [9,11,14] and organ-protective effects of sevoflurane have been suggested [23,24]. In this regard, further investigations on the long-term application of inhalative anesthetics on intensive care units may help to emerge novel sedation concepts employing these agents that may thus possibly help to provide increased safety for the patients cared for. Regarding a benefit-to-risk analysis, it may be estimated that the benefits of applying sevoflurane may outweigh the possible risks (side effects) and the prospects of conventional intravenous sedation concepts.

The consent procedure comprises the practice of the possibility of consent by a previously appointed legal representative as well as the primary or ongoing consent of the patient himself.

## Discussion

It has already been shown in the late 1980s that the application of isoflurane in intensive care treatment of certain groups of patients (that is, patients that are difficult to sedate or with bronchial asthma) offers distinct advantages compared to intravenous sedation [6,9]. Korth *et al*. examined 20 mechanically ventilated patients after sedation with isoflurane (2 to 27 days) and demonstrated that in addition to an improved sedation quality and a reduction of bronchial spasticity there were no clinically relevant changes in serum fluoride concentration or other organ-related side effects [6]. In these studies, the anesthesia gas was either applied via the usual vapor in connection with a ventilation device (Servo 900C) or by using a ‘closed anesthesia system’ (Physioflex®, Zeus®, Dräger, Lübeck, Germany).

Because of the short availability of the system so far, clinical experience with the AnaConDa® system is currently limited with most studies referring to the substance isoflurane. For example, Sakey *et al*. demonstrated that the time until extubation was distinctly shorter after sedation with isoflurane compared to sedation with midazolam following an application time up to 96 h (isoflurane: 10 ± 5 min *vs*. midazolam: 250 ± 270 min) [9]. However, further improvement of the concept of ‘inhalative sedation’ applied in critically ill patients could be expected from application of sevoflurane as it provides favorable pharmacokinetic as well as possibly organ-protective effects [8,9,25].

Following inhalative application, 3% to 6% of sevoflurane is metabolized and metabolic products such as fluoride ions may exert toxic effects that have to be taken into account considering long-term use of sevoflurane. Although previous studies revealed an increase of the measured fluoride concentration already shortly after the beginning of application, evidence for clinical relevance of fluoride ion exposition even after long-time application of sevoflurane could not be provided [24,26].

Despite previous studies, the application of sevoflurane for long-term sedation in critically ill patients regarding patient safety and quality of sedation has not been investigated so far. As this group of patients confers significant morbidity and mortality, strategies to improve the quality of care and outcome of these patients is therefore especially needed.

## Conclusion

In conclusion, the present clinical study is powered to test the hypothesis that sevoflurane is non-inferior to conventional intravenous sedation with propofol in the context of long-term sedation of critically ill patients and moreover associated with a better sedation quality without any detrimental side effects in this patient population.

### Trial status

The trial is currently ongoing; patient recruitment shall be completed in 2012.

## Competing interests

JS has received a speakers’ fee and reimbursement of expenses for scientific lectures referring to analgosedation as well as inhalative sedation of intensive care patients from Abbott GmbH & Co KG (Wiesbaden, Germany), Sedana Medical (Uppsala, Sweden), and Baxter (Unterschleißheim, Germany). All other authors have no conflicts of interest.

## Authors’ contributions

JS is the principal investigator of the study and responsible for the conception, protocol design, and organization of the financial support. JS submitted and received the financial support by the Wilhelm-Roux-Program of the Medical Faculty of the University of Halle to initiate this clinical trial. AW has provided statistical guidance, is responsible for the estimation of the sampling size and final statistical analysis. JS and PK are involved in the management of the study and responsible for data acquisition and study coordination. AS and PK wrote and NMW revised the manuscript. All authors have approved the final version and submission of the present paper to *Trials*. All authors read and approved the final manuscript.

## References

[B1] Martin J Heymann A Bäsell K Baron R Biniek R Bürkle H Dall P Dictus C Eggers V Eichler I Engelmann L Garten L Hartl W Haase U Huth R Kessler P Kleinschmidt S Koppert W Kretz FJ Laubenthal H Marggraf G Meiser A Neugebauer E Neuhaus U Putensen C Quintel M Reske A Roth B Scholz J Schröder S Evidence and consensus-based German guidelines for the management of analgesia, sedation and delirium in intensive care--short version Ger Med Sci 2010 8 Doc02 20200655 10.3205/000091 PMC2830566

[B2] Badeva B Iskrenova I Panchev P Gerov I Nemtsova D Radichkova V Petrov P Gerzilova L Aluan K Prolonged propofol (Diprivan) infusion for sedation in the critically ill Khirurgiia 1996 49 28 30 8968139

[B3] Darrouj J Karma L Arora R Cardiovascular manifestations of sedatives and analgesics in the critical care unit Am J Ther 2009 16 339 353 10.1097/01.pap.0000249925.76324.47 19092649

[B4] Kelly DF Propofol-infusion syndrome J Neurosurg 2001 95 925 926 10.3171/jns.2001.95.6.0925 11765835

[B5] Ostermann ME Keenan SP Seiferling RA Sibbald WJ Sedation in the intensive care unit: a systematic trial JAMA 2000 Suppl 11 1451 1459 10732935 10.1001/jama.283.11.1451

[B6] Korth M Opitz A Opitz A Methoden der Analgosedierung in der Intensivmedizin Erste klinische Erfahrungen in der Langzeitsedierung mit Isofluran 1989 Braunschweig: Bethel-Verlag 139 146

[B7] Yates DW Hughes JA Isoflurane and long-term sedation Anaesthesia 1993 48 267 268 8384805 10.1111/j.1365-2044.1993.tb06920.x

[B8] Röhm KD Mengistu A Boldt J Mayer J Beck G Piper SN Renal integrity in sevoflurane sedation in the intensive care unit with the anesthetic-conserving device: a comparison with intravenous propofol sedation Anesth Analg 2009 Suppl 6 1848 1854 10.1213/ane.0b013e3181a1988b 19448211

[B9] Sackey PV Martling CR Granath F Radell PJ Prolonged isoflurane sedation of intensive care unit patients with the Anesthetic Conserving Device Crit Care Med 2004 32 2241 2246 15640636 10.1097/01.ccm.0000145951.76082.77

[B10] Breen D Karabinis A Malbrain M Morais R Albrecht S Jarnvig IL Parkinson P Kirkham AJ Decreased duration of mechanical ventilation when comparing analgesia-based sedation using remifentanil with standard hypnotic-based sedation for up to 10 days in intensive care unit patients: a randomized trial [ISRCTN47583497] Crit Care 2005 Suppl 3 R200 R210 15987391 10.1186/cc3495 PMC1175879

[B11] Soukup J Schärff K Kubosch K Pohl C Bomplitz M Kompardt J State of the art: sedation concepts with volatile anesthetics in critically Ill patients J Crit Care 2009 Suppl 4 535 544 19327951 10.1016/j.jcrc.2009.01.003

[B12] Mesnil M Capdevila X Bringuier S Trine PO Falquet Y Charbit J Roustan JP Chanques G Jaber S Long-term sedation in intensive care unit: a randomized comparison between inhaled sevoflurane and intravenous propofol or midazolam Intensive Care Med 2011 Suppl 6 933 941 21445642 10.1007/s00134-011-2187-3

[B13] Stuttmann R Pillukat T Müller-Gorges M Knüttgen D Doehn M Opitz A Methoden der Analgosedierung auf der Intensivstation Sedierung mit Isofluran unter pulmonalen und hämodynamischen Aspekten 1989 Braunschweig: Bethel-Verlag 139 146

[B14] Tanigami H Yahagi N Kumon K Watanabe Y Haruna M Matsui J Hayashi H Long-term sedation with isoflurane in postoperative intensive care in cardiac surgery Artif Organs 1997 Suppl 1 21 23 9012901 10.1111/j.1525-1594.1997.tb00693.x

[B15] Meiser A Laubenthal H Inhalational anaesthetics in the ICU: theory and practice of inhalational sedation in the ICU, economics, risk-benefit Best Pract Res Clin Anaesthesiol 2005 Suppl 3 523 538 16013698 10.1016/j.bpa.2005.02.006

[B16] Meiser A Bellgardt M Vogelsang H Sirtl C Weber T Functioning of the anaesthetic conserving device: aspects to consider for use in inhalational sedation Anaesthesist 2010 Suppl 11 1029 1040 20878139 10.1007/s00101-010-1779-6

[B17] Perhag L Reinstrup P Thomasson R Werner O The Reflector: a new method for saving anaesthetic vapours Br J Anaesth 2000 Suppl 3 482 486 11103198 10.1093/bja/85.3.482

[B18] Thomasson R Luttropp H Werner O A reflection filter for isoflurane and other anaesthetic vapours Eur J Anaesthesiol 1989 6 89 94 2721507

[B19] Muellejans B Matthey T Scholpp J Schill M Sedation in the intensive care unit with remifentanil/propofol versus midazolam/fentanyl: a randomized, open-label, pharmacoeconomic trial Crit Care 2006 Suppl 3 R91 16780597 10.1186/cc4939 PMC1550941

[B20] European Medicines A ICH Harmonised Tripartite Guideline E6: Note for Guidance on Good Clinical Practice (PMP/ICH/135/95) 2002 London: European Medicines Agency

[B21] Bundesdatenschutzgesetz (BDSG) vom 20. Dezember 1990 (BGBl. I S. 2954) neugefasst durch Bekanntmachung vom 14. Januar 2003 (BGBl. I S. 66), zuletzt geändert durch Gesetz vom 29.07.2009 (BGBl. I, S. 2254), durch Artikel 5 des Gesetzes vom 29.07 2009 BGBl. I, S. 2355 [2384] und durch Gesetz vom 14.08.2009 (Federal Law Gazette I, S. 2814

[B22] Medicinal Products Actin the version published on 12 December 2005 (Federal Law Gazette [BGBl.]) Part I p. 3394, last amended by Article 1 of the Ordinance of 28 September 2009 Federal Law Gazette I p. 3172

[B23] Kehl F Smul TM Lange M Redel A Roewer N Organprotektion durch volatile Anästhetika Anästhesiologie und Intensivmedizin 2004 11 491 507

[B24] Röhm KD Wolf MW Schöllhorn T Schellhaass A Boldt J Piper SN Short-term sevoflurane sedation using the Anaesthetic Conserving Device after cardiothoracic surgery Intensive Care Med 2008 Suppl 9 1683 1689 10.1007/s00134-008-1157-x 18500419

[B25] Meiser A Sirtl C Bellgardt M Lohmann S Garthoff A Kaiser J Hügler P Laubenthal HJ Desflurane compared with propofol for postoperative sedation in the intensive care unit Br J Anaesth 2003 Suppl 3 273 280 12594136 10.1093/bja/aeg059

[B26] Migliari M Bellani G Rona R Isgrò S Vergnano B Mauri T Patroniti N Pesenti A Foti G Short-term evaluation of sedation with sevoflurane administered by the anesthetic conserving device in critically ill patients Intensive Care Med 2009 Suppl 7 1240 1246 19189080 10.1007/s00134-009-1414-7

